# How Workplace Telepressure Fuels Job Burnout Among Educators: Mediated by Work-Related Rumination and Moderated by Perceived Organizational Support

**DOI:** 10.3390/bs15081109

**Published:** 2025-08-16

**Authors:** Ke Qin, Ze Yu, Qihai Cai, Nan Jiang, Kin San Chung

**Affiliations:** 1 School of Information and Electronic Engineering, Shandong Technology and Business University, Yantai 264005, China; 2 School of Business, Macau University of Science and Technology, Macau SAR 999078, China; 3 University International College, Macau University of Science and Technology, Macau SAR 999078, China; 4 Faculty of Humanities and Social Sciences, City University of Macau, Macau SAR 999078, China

**Keywords:** workplace telepressure, job burnout, work-related rumination, perceived organizational support

## Abstract

The rapid advancement in information and communication technologies (ICT) has improved workplace productivity but also introduced new psychosocial stressors. Workplace telepressure, the urge to respond quickly to work-related messages sent via digital communication tools, often erodes employees’ psychological well-being and blurs work–life boundaries. This study, grounded in the Job Demands–Resources (JD-R) model, investigates the influence mechanism between workplace telepressure and job burnout. Using data collected from 323 middle school teachers in China, results revealed that workplace telepressure significantly predicted job burnout. In addition, work-related rumination partially mediated this relationship, indicating a cognitive mechanism linking telepressure and burnout. Furthermore, perceived organizational support moderated the relationship between work-related rumination and job burnout. Specifically, when perceived support was high, the impact of rumination on burnout was weakened. These findings provide theoretical insights and practical implications for fostering healthy work environments and promoting psychological well-being in the digital era.

## 1. Introduction

With the rapid development of the digital economy and continuous advances in information and communication technologies (ICT), digital communication tools have become integral to nearly every profession. The widespread use of smartphones, laptops, and collaborative software has enabled employees to engage in work-related communication across both physical and temporal boundaries (Farivar et al., 2024). This transformation has been further accelerated by the COVID-19 pandemic, during which “cloud platforms” and virtual meetings normalized remote connectivity and redefined traditional workplace practices (Aleem et al., 2023; Karl et al., 2022).

Information and communication technologies offer notable advantages, such as increased flexibility, autonomy, and perceived work efficiency (Leung, 2011). Employees can complete tasks from remote locations, reducing commuting time and enhancing productivity, which can improve job satisfaction and engagement (Ragsdale & Hoover, 2016; Ter Hoeven et al., 2016). However, these technological affordances come at a psychological cost. Scholars have identified a “double-edged sword” effect, where information and communication technologies facilitate constant connectivity but simultaneously blur the boundaries between work and personal life (Dén-Nagy, 2014). This perpetual accessibility often cultivates an “always-on” culture, leading to increased stress, disturbed work–life balance, and work–family conflict (Dettmers et al., 2016; Park et al., 2018).

Workplace telepressure, defined as the urge to respond promptly to work-related messages via digital devices (Barber & Santuzzi, 2015), presents a common psychological stressor in the modern workplace. Unlike proactive communication, workplace telepressure stems from perceived obligation and psychological compulsion, which can gradually deplete cognitive and emotional resources, ultimately leading to increased job burnout (Barber & Santuzzi, 2017; Budnick et al., 2020). In education settings, where communication and administrative coordination often extend beyond work hours, teachers are especially susceptible to such pressures. Schools increasingly rely on digital tools to maintain instructional and organizational workflows, resulting in teachers being frequently required to remain digitally connected. This hyper-connectivity heightens workload demands and often leads to work-related rumination, wherein individuals mentally revisit unfinished tasks during off-hours, further impairing recovery and well-being.

Grounded in the Job Demands–Resources (JD-R) model (Bakker et al., 2023), this study examines the influencing mechanism between workplace telepressure and job burnout. The JD-R model highlights how increased job demands can drain personal resources and result in burnout, while supplementing job resources may buffer such effects. The JD-R model is particularly suitable for exploring the burnout mechanisms in the education sector, where the resource–recovery balance is often disrupted by extended digital engagement. In China, the rapid development of smart education and Education 4.0 initiatives has accelerated the integration of digital technologies in teaching. These reforms have increased teachers’ reliance on digital platforms, leading to heavier communication demands and extended working hours beyond the classroom (Mukul & Büyüközkan, 2023). As a result, Chinese educators are particularly vulnerable to workplace telepressure, making it important to explore its impact in this context.

This study proposes that workplace telepressure influences teacher burnout through the mediating role of work-related rumination. Furthermore, the study examines perceived organizational support as a key boundary condition that may buffer this effect. By investigating these mechanisms, the study contributes to current discourse on how to foster a healthy work environment that promotes employee well-being and job satisfaction, especially in the increasingly digitalized education sector.

## 2. Literature Review and Hypothesis Materials

### 2.1. Workplace Telepressure and Job Burnout

Workplace telepressure is the psychological impulse of an individual to prioritize work information and respond quickly when using communication devices during work. Employees who are exposed to higher levels of workplace telepressure have poorer sleep quality and life satisfaction, and they usually perceive higher levels of burnout and stress (Barber & Santuzzi, 2017). For example, current office communication software typically indicates message status as “read” after viewing, which conditions employees to maintain constant availability for immediate digital responses. According to the JD-R model, excessive job demands will cause the consumption of physical and mental resources for individuals. Being in this state for a long time leads to employees having a hard-to-eliminate sense of tension and anxiety regarding the use of electronic tools for communication and work, and individual resources are constantly consumed and difficult to recover (Cambier et al., 2019; Hobfoll, 2001). In the context of education, such digital communication demands are particularly significant. Chou and Chou (2021) found that technostress significantly predicts teachers’ intention to continue using digital platforms for online instruction, highlighting the psychological burden caused by persistent connectivity. Similarly, Imran et al. (2025) emphasized that the post-COVID-19 digital transformation in schools has intensified workload expectations and blurred work–life boundaries, resulting in elevated emotional exhaustion among teachers. Moreover, workplace telepressure leads teachers to pay excessive attention to the dynamics occurring in the workplace, forcing them to pay less attention to family, personal life, and other aspects. This is bound to cause individuals to feel powerless and negative about their work, thereby triggering job burnout. Furthermore, workplace telepressure may cause fragmented attention in employees. Under such circumstances, individuals often engage in continuous task-switching and multitasking, which elevates cognitive load and psychological pressure (Barley et al., 2011). Over time, such demands may induce technostress that contributes to increased work fatigue and negative emotional states (Sonnentag et al., 2018; Chang et al., 2024). When employees struggle to manage simultaneous responsibilities, they often experience a diminished sense of control over their work, which may reinforce feelings of helplessness and exacerbate job burnout. Therefore, the following hypothesis is proposed:

**Hypothesis** **1.** *Workplace telepressure is positively related to job burnout.*

### 2.2. Mediating Role of Work-Related Rumination

Workplace telepressure increases employees’ workload and extends their cognitive engagement into non-working hours. Employees often find themselves involuntarily reflecting on work-related issues during leisure time. This repetitive thinking of work-related issues may manifest in two forms of work-related rumination: affective rumination, which is emotionally distressing, and problem-solving pondering, which is cognitively engaging when the tasks are perceived as solvable or meaningful (Cropley & Zijlstra, 2011). The rise in ICT usage has further intensified this dynamic. For example, responding to work-related emails after work triggers work-related rumination to undermine employee well-being at bedtime (Minnen et al., 2021). This constant connectivity elevates job demands, drains psychological energy, and fosters negative emotional experiences to increase the likelihood of affective rumination (Park et al., 2018). Moreover, telepressure can stimulate problem-solving pondering during leisure time, especially when individuals feel pressure to resolve unfinished tasks quickly (Cambier & Vlerick, 2022). In both cases, the inability to detach mentally from work hinders psychological recovery and threatens overall well-being (Hu et al., 2019). This tendency is particularly pronounced in the education sector. Teachers are especially prone to work-related rumination due to the emotionally demanding and highly personalized nature of their work (Türktorun et al., 2020). Workplace telepressure prompts individuals to process work-related electronic information in real time to actively and continuously think about work-related issues and improvement practices after work. Consequently, workplace telepressure has a positive impact on work-related rumination.

Additionally, work-related rumination has been identified as a key cognitive–emotional process contributing to job burnout (Košir et al., 2015). While affective rumination tends to intensify emotional strain and predict higher levels of burnout (Vandevala et al., 2017; Sousa & Neves, 2021), problem-solving pondering may sometimes reduce burnout by promoting cognitive engagement and coping (Bisht, 2019; Mullen et al., 2020). The persistent rumination—particularly when emotionally charged—often elevates emotional burden and cognitive strain, aggravating job burnout over time (Saloviita & Pakarinen, 2021). Moreover, when individuals are unable to mentally disengage from work, their cognitive and emotional systems remain in a heightened state of activation, which gradually prolongs stress responses, drains psychological resources, and delays recovery (Haun & Baethge, 2020). Over time, the accumulation of such unresolved strain can lead to emotional exhaustion, a reduced sense of accomplishment, and ultimately, job burnout. Therefore, work-related rumination has a positive impact on job burnout.

To sum up, individuals experiencing high levels of telepressure often find it difficult to mentally disengage from work during non-working hours, which increases their tendency to ruminate about unresolved tasks (Pfaffinger et al., 2022; Santuzzi & Barber, 2018). Work-related rumination, in turn, disrupts psychological detachment and recovery, leading to fatigue, emotional distress, and reduced life satisfaction (Blanco-Encomienda et al., 2020). Persistent reflection on job demands, especially when emotionally charged, can exacerbate employees’ psychological burden and increase their vulnerability to burnout (Allwood et al., 2022; He et al., 2020). In this regard, work-related rumination may play a mediating role between workplace telepressure and job burnout. According to the JD-R model, workplace telepressure presents a job demand that compels individuals to remain hyper-connected via digital tools, even after working hours; this continuous connection triggers work-related rumination that depletes both cognitive and emotional resources, making it difficult for them to fully recover; this mental over-engagement often exacerbates fatigue, reduces work efficiency, and increases irritability and anxiety—factors that cumulatively contribute to job burnout. Taken together, workplace telepressure triggers work-related rumination, leading to job burnout. Thus, we propose that:

**Hypothesis** **2.** *Work-related rumination mediates the relationship between workplace telepressure and job burnout.*

### 2.3. Moderating Role of Perceived Organizational Support

According to the JD-R model, increased job demands often drain personal resources and produce negative outcomes, while supplementing extra job resources may mitigate the detrimental effects. With the pervasive use of ICT tools in the workplace, persistent work-related rumination depletes personal psychological resources to intensify psychological fatigue and emotional exhaustion, calling for supplementary job resources to meet the job demands.

Perceived organizational support is conceptualized as employees’ general belief that their organization recognizes their efforts and is genuinely concerned about their well-being, development, and job performance. Higher levels of perceived organizational support are associated with increased job satisfaction and a reduced likelihood of turnover (Kurtessis et al., 2017). Perceived organizational support significantly lowers nursing professionals’ emotional exhaustion (Lartey et al., 2021). Similarly, teachers who reported greater organizational support were less likely to suffer from burnout symptoms (Lavian, 2012). Perceived organizational support, as a psychological resource, may mitigate the detrimental effects. Specifically, when employees perceive their organization as supportive, they are less susceptible to negative emotional responses—such as anxiety or guilt—stemming from work-related setbacks. Perceived organizational support reduces fear of blame and enables more adaptive emotion regulation (Luthans et al., 2008). Individuals with higher levels of perceived organizational support are better able to maintain task engagement and protect themselves against burnout. Supporting this notion, He et al. (2021) demonstrated that perceived organizational support mitigated the adverse influence of negative rumination on job burnout. This buffering effect is likely driven by the satisfaction of key socioemotional needs, including being valued, respected, and emotionally supported, which collectively dampen feelings of distress and exhaustion.

Consequently, perceived organizational support, as a supplementary psychological resource, reduces the detrimental effects of work-related rumination on job burnout. Therefore, the following hypothesis is proposed:

**Hypothesis** **3.** *Perceived organizational support moderates the relationship between work-related rumination and job burnout, such that this relationship will be weaker when perceived organizational support is high than when it is low.*

**Figure 1:**
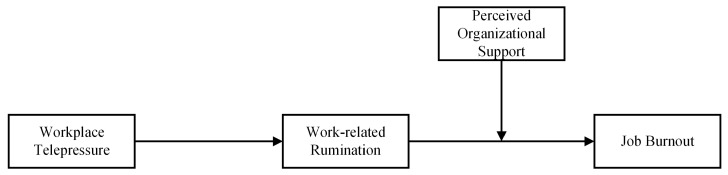
Proposed moderated mediation model.

Integrating the above-mentioned mediated and moderated relationships, we propose a moderated mediation model (Figure 1), such that the indirect effects of workplace telepressure on job burnout via rumination will be contingent upon perceived organizational support. According to the JD-R model, individuals experiencing the job demands of telepressure often find it difficult to mentally disengage from work-related issues during non-work hours, depleting their psychological resources to ruminate about unresolved tasks. Then, work-related rumination disrupts their psychological detachment and recovery, leading to job burnout. Perceived organizational support supplements extra psychological resources, helping them to relieve cognitive exhaustion and accelerate the recovery process. More specifically, perceived organizational support, as a job resource, is likely to mitigate the effects of workplace telepressure on job burnout through work-related rumination. Therefore, we propose that:

**Hypothesis** **4.** *Perceived organizational support moderates the indirect effect of workplace telepressure on job burnout via work-related rumination, such that this relationship will be weaker when perceived organizational support is high than when it is low.*

## 3. Research Methods

### 3.1. Sampling and Data Collection

This study mainly surveyed middle school teachers in regions such as Shandong, Anhui, Fujian, and Guangdong, and distributed electronic questionnaires to them to obtain information. The participants were informed of the intention of this study before answering the questions. They were asked to participate in the survey anonymously to guarantee the confidentiality of their personal information. Prior to the formal questionnaire distribution, this study conducted a pilot survey with a small cohort of teachers in Shandong Province to evaluate the appropriateness of item wording and layout design. Upon confirming full validity through their feedback, the official questionnaire was formally distributed.

**Figure 2:**
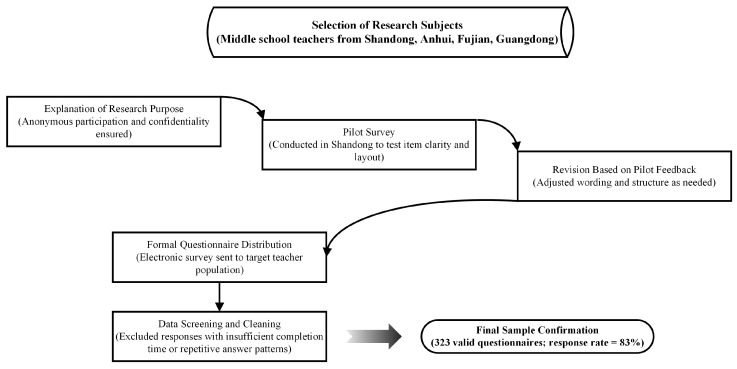
Data collection and sample selection process.

A total of 388 questionnaires were distributed. Invalid answers with problems such as insufficient answering time or choosing the same option for most questions were excluded. As a result, 323 valid sample data were obtained, with an effective response rate of 83%. The detailed procedure for data collection is illustrated in Figure 2. The sample consisted of 61.6% female and 38.4% male participants. Most respondents were aged 26–55, with the largest proportions in the 36–45 and 46–55 age groups (each 27.6%). Teaching experience varied, with 32.8% having 0–5 years and 31.9% over 20 years. The majority held a bachelor’s degree (83.3%), followed by a master’s (13.9%). Regarding school type, 57.9% of participants worked in senior high schools, while 42.1% worked in junior high schools.

### 3.2. Measures

The variables involved in this study were measured using well-established scales with demonstrated psychometric validity. The study used a five-point Likert-type scale to measure all variables, with ratings ranging from 1 (strongly disagree) to 5 (strongly agree).

Workplace telepressure was assessed via a six-item scale developed by Barber and Santuzzi (2015), which captures employees’ psychological compulsion to respond quickly to work-related electronic communications. Respondents evaluated items such as “It is hard for me to concentrate on other activities when I receive work-related messages.” The Cronbach’s alpha for this scale was 0.81.

Job burnout was measured using the Maslach Burnout Inventory–Educators Survey (Maslach & Jackson, 1981), consisting of 22 items categorized into emotional exhaustion, depersonalization, and reduced personal accomplishment. A sample item is “I feel physically and emotionally drained from my work,” and responses were provided on the same five-point scale. The Cronbach’s alpha for this scale was 0.88.

Work-related rumination was measured through a 10-item scale developed by Cropley et al. (2012), which distinguishes between affective rumination and problem-solving pondering. Participants indicated their agreement with items such as “I feel tense during my free time because I keep thinking about work.” The Cronbach’s alpha for this scale was 0.87.

Perceived organizational support was measured using the eight-item instrument developed by Eisenberger et al. (1997), which evaluates the extent to which individuals perceive their organization as valuing their contributions and caring for their well-being. The Cronbach’s alpha for this scale was 0.88.

To account for potential confounding effects, this study included gender, age, teaching tenure, and school type as control variables.

### 3.3. Data Analysis

The statistical analyses were performed using SPSS 26.0 and AMOS 26.0. Internal consistency for each construct was assessed using Cronbach’s alpha coefficients. A confirmatory factor analysis (CFA) was performed in this study to assess the construct validity of the measurement model. Convergent and discriminant validity were examined, and model fit was evaluated using χ^2^/df, RMSEA, CFI, TLI, and IFI indices. Pearson correlation coefficients were computed to examine the bivariate associations among workplace telepressure, work-related rumination, perceived organizational support, and job burnout.

To test the hypothesized mediation model, PROCESS macro (Model 4) with 5000 bias-corrected bootstrap samples was employed. The analysis followed a three-step procedure (Baron & Kenny, 1986): (1) regressing job burnout on workplace telepressure, (2) regressing work-related rumination on workplace telepressure, and (3) regressing job burnout on both workplace telepressure and work-related rumination. The significance of indirect effects was determined by inspecting 95% bootstrap confidence intervals. To test the moderating effect of perceived organizational support on the relationship between work-related rumination and job burnout, PROCESS macro (Model 1) was applied. Prior to conducting the moderation analysis, all continuous variables were mean-centered to reduce potential multicollinearity and to ensure accurate interpretation of interaction effects. Where a significant interaction was detected, simple slope analyses were conducted at ±1 standard deviation of perceived organizational support to probe conditional effects. The use of bootstrapped confidence intervals enhanced the robustness of inference, and effects were considered statistically significant if the 95% confidence interval did not include zero.

## 4. Results

### 4.1. Measurement Model

We adopted the packaged item method of factor loading and conducted discriminative validity tests on four variables, namely workplace telepressure, work-related rumination, perceived organizational support, and job burnout, through AMOS 26.0 data analysis software. The result of the four-factor model (χ^2^/df = 3.28, RMSEA = 0.08, IFI = 0.96, TLI = 0.95, CFI = 0.96) is demonstrated in Table 1. Compared with all alternative models, the four-factor model provides the best fit to the data. Specifically, IFI, TLI, and CFI are all greater than 0.9, and RMSEA is less than 0.1, indicating that the discriminant validity of the four variables in this study is good and meets the standards.

### 4.2. Descriptive Statistics and Correlations

This study conducted a correlation analysis of each variable, and the results are shown in Table 2 as follows:

Table 2 shows that workplace telepressure was significantly and positively correlated with job burnout (r = 0.25, *p* < 0.01). Workplace telepressure was significantly and positively correlated with work-related rumination (r = 0.33, *p* < 0.01). Work-related rumination was significantly and positively correlated with job burnout (r = 0.24, *p* < 0.01). The results provide preliminary empirical support for the hypotheses proposed earlier in this study.

### 4.3. Hypothesis Testing

As displayed in Model 1 of Table 3, workplace telepressure was positively associated with job burnout (β = 0.19, *p* < 0.001), which supports Hypothesis 1. Second, workplace telepressure was positively related to work-related rumination (β = 0.31, *p* < 0.001, Model 2), and work-related rumination was positively related to job burnout (β = 0.19, *p* < 0.001, Model 3), meeting the prerequisites for testing the mediation. Third, we added workplace telepressure and work-related rumination in the regression model simultaneously (see Table 3 Model 4). The relationship between work-related rumination and job burnout remained significant (β = 0.14, *p* < 0.01), and the regression coefficient of workplace telepressure decreased (from β = 0.19, *p* < 0.001 to β = 0.15, *p* < 0.001), indicating partial mediation. Moreover, we applied the bootstrapping method, resampling 5000 times, to examine the indirect effect of workplace telepressure on job burnout via work-related rumination. As indicated in Table 4, the bootstrapping results validated the significance of the indirect effects (95% CI [0.01, 0.08]), thus supporting Hypothesis 2.

**Figure 3:**
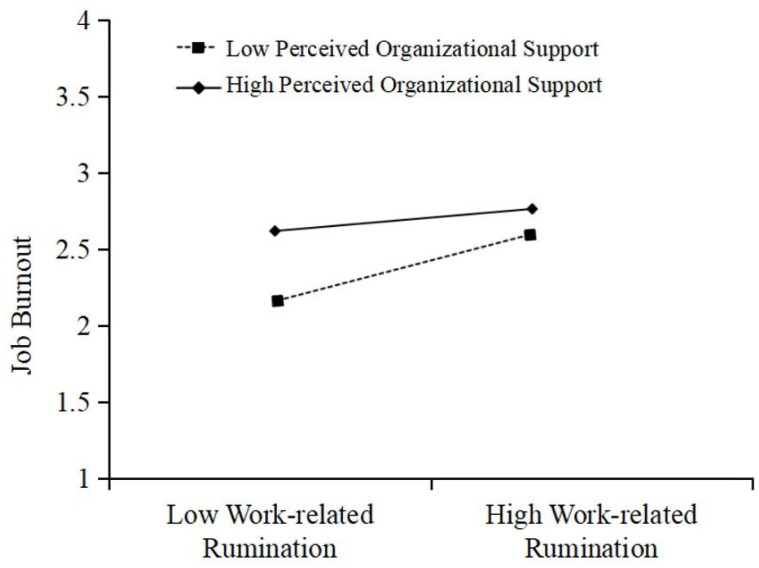
Moderating results of perceived organizational support on the relationship between work-related rumination and job burnout.

Hypothesis 3 proposed that perceived organizational support would moderate the relationship between work-related rumination and job burnout. As indicated in Model 5 of Table 3, the interaction between work-related rumination and perceived organizational support was statistically significant (β = −0.12, *p* < 0.01), suggesting that perceived organizational support negatively moderates the relationship between work-related rumination and job burnout. To further probe this interaction, a conditional effect analysis was conducted using simple slopes at high (+1 SD) and low (−1 SD) levels of perceived organizational support. As shown in Table 5 and illustrated in Figure 3, the effect of work-related rumination on job burnout was stronger with low perceived organizational support (effect = 0.32, 95% CI [0.21, 0.42]) compared to high perceived organizational support (effect = 0.11, 95% CI [0.00, 0.23]). These findings suggest that perceived organizational support acts as a buffering mechanism that weakens the effects of work-related rumination on job burnout.

To examine the moderated mediation model, we tested the conditional indirect effect of workplace telepressure on job burnout through work-related rumination at different levels of perceived organizational support (mean ± 1 SD). As indicated in Table 6, the indirect effect was found to be significant (effect = 0.08, 95% CI [0.03, 0.14]) for lower levels of perceived organizational support but insignificant for higher levels of perceived organizational support (effect = 0.02, 95% CI [−0.01, 0.06]). The indirect effect of workplace telepressure on job burnout via work-related rumination is stronger when perceived organizational support is low as opposed to high, thus supporting Hypothesis 4.

## 5. Discussion

Grounded in the JD-R model, this study explores the influence mechanism between workplace telepressure and job burnout, examining the mediating role of work-related rumination and the moderating role of perceived organizational support. Using data collected from 323 middle school teachers in China, the results indicate that workplace telepressure significantly increased teachers’ job burnout. Additionally, work-related rumination partially mediated this relationship, suggesting that the cognitive and emotional strain caused by continuous work-related thoughts increases burnout. Furthermore, perceived organizational support negatively moderates the effect of work-related rumination on job burnout and the indirect effect of workplace telepressure on job burnout through work-related rumination.

### 5.1. Theoretical Contributions

First, this study focused on middle school teachers as the primary sample group and investigated the mechanisms underlying their experience of job burnout. Prior literature has systematically analyzed the antecedents of teacher burnout across individual (e.g., teaching tenure), organizational (e.g., school type), and societal (e.g., policy environment) levels. However, the role of digitally induced stressors, such as workplace telepressure—a relatively novel stressor—has received limited attention in the educational domain. This study enriches the existing discourse on burnout by introducing a job-level demand that has become increasingly salient in the digital communication era.

Second, this study extends the application of the JD-R model by identifying work-related rumination as a mediator linking telepressure to burnout. Although prior studies have established that rumination impedes resource recovery (Van Laethem et al., 2019) and hampers job performance (Baranik et al., 2017), its bridging role in the telepressure–burnout pathway has not been sufficiently explored. This study empirically confirms that persistent cognitive engagement with work-related content during non-work hours exacerbates emotional exhaustion, thus unveiling a key psychological mechanism through which telepressure depletes individual well-being.

Third, our findings concerning the mediating role of work-related rumination are consistent with those of Türktorun et al. (2020), who highlighted that teachers often struggle to achieve psychological detachment due to the emotionally demanding nature of their profession. Likewise, the observed positive correlation between workplace telepressure and job burnout supports Saloviita and Pakarinen (2021), who identified student-related and organizational demands as primary burnout sources in educational settings. This study is an empirical extension grounded in well-established educational psychology theories—particularly in response to emerging digital stressors within teaching contexts.

Last, perceived organizational support, as a boundary condition, moderates the link between rumination and burnout. When teachers perceive strong organizational support, the detrimental influence of work-related rumination on burnout is substantially mitigated. Fostering a supportive work climate, digital leadership, for example, can enhance employee resilience and reduce burnout in digitally demanding work settings (Cai et al., 2024). This aligns with the JD-R model that providing more job resources can buffer the adverse effects of job demands.

### 5.2. Practical Implications

Based on the research findings, this study provides three practical implications. First, certain measures should be taken to build a positive mindset for teachers to reduce the emotional exhaustion caused by telepressure. For example, fostering teachers’ proactive career orientation enhances their career adaptability (Chang et al., 2023). With enhanced career adaptability in the digital era, teachers can restore their personal resources outside of working hours and face the next day’s work with full energy, thus creating a virtuous cycle.

Second, mitigating teacher burnout under digital pressures requires optimized communication structures. Schools should adopt formal digital communication norms to limit non-essential work-related contact outside working hours. Practices such as establishing “quiet periods” (e.g., after 18:00 or on weekends), inspired by “right to disconnect” policies implemented in regions like Queensland, Australia, can support psychological detachment and reduce cognitive overload. Moreover, consolidating communication through centralized platforms (e.g., DingTalk, WeCom) and disabling off-hour push notifications can alleviate technostress and mitigate its impact on emotional exhaustion.

Third, schools should enhance the perceived organizational support for teachers. Teachers who receive high-level perceived organizational support can adapt to and adjust to the adverse effects of workplace telepressure in a timely manner. Higher perceived institutional support significantly reduces technostress among educators (Chou & Chou, 2021). Well-structured digital management policies helped teachers reduce their psychological burden and maintain emotional balance (Imran et al., 2025). Offering more job resources, such as perceived fairness and a supportive climate, is an important basis for teachers to form a sense of organizational support (Chang et al., 2025), thus reducing job burnout.

### 5.3. Limitations and Future Directions

While this study enriches our understanding of workplace telepressure and its psychological consequences among middle school teachers, three limitations must be acknowledged. First, the sample in this study, although spanning several provinces in China, was not geographically balanced. A substantial proportion of respondents were from Shandong Province, and the sample had a gender imbalance, with male participants underrepresented—an issue common in the education sector. These sampling constraints may have introduced bias or limited the applicability of the findings to broader teacher populations. Future studies are encouraged to adopt more stratified sampling strategies to enhance representativeness.

Second, while the current model effectively demonstrates the mediating role of work-related rumination, it does not differentiate between its two key subtypes: affective rumination and problem-solving pondering. Given their opposing effects on psychological outcomes, this aggregation may obscure important differences in how each subtype contributes to burnout. Future research should consider modeling these dimensions separately, as doing so could offer deeper insights into the emotional and cognitive processes linking workplace telepressure and job burnout.

Third, the study employed a cross-sectional research design, which restricts causal inference. Although the hypothesized relationships are grounded in established theory, longitudinal or experimental studies are needed to validate the directionality and stability of these effects over time.

## 6. Conclusions

By identifying the relationships between workplace telepressure and job burnout among middle school teachers, this study contributes to the advancement of research on workplace telepressure. Our findings, grounded in the JD-R model, highlight the impact of workplace telepressure on job burnout through work-related rumination, with perceived organizational support moderating the process. Organizations should implement strategies to minimize telepressure and support employees’ psychological well-being to foster a healthier work environment.

## Figures and Tables

**Table 1:** Results of confirmatory factor analyses.

Measurement Model	χ^2^	df	χ^2^/df	IFI	TLI	CFI	RMSEA
The hypothesized four-factor model	157.33	48	3.28	0.96	0.95	0.96	0.08
Three-factor model (combining WR and JB)	710.07	51	13.92	0.79	0.72	0.78	0.20
Two-factor model (combining WR, POS, and JB)	2018.55	53	38.09	0.36	0.20	0.36	0.34
One-factor model (combining WT, WR, POS, and JB)	2356.84	54	43.65	0.25	0.08	0.25	0.36

Note: *N* = 323; WT = workplace telepressure; WR = work-related rumination; POS = perceived organizational support; JB = job burnout.

**Table 2:** Descriptive statistics and correlations.

Variable	M	SD	1	2	3	4	5	6	7
Gender	0.59	0.49							
Age	2.72	1.09	−0.01						
Teaching age	2.78	1.34	0.00	0.88 **					
School type	0.58	0.49	0.03	−0.10	−0.05				
Workplace telepressure	3.74	0.77	0.04	−0.01	0.00	0.04			
Work-related rumination	3.50	0.72	−0.04	−0.06	−0.07	0.02	0.33 **		
Perceived organizational support	2.73	0.89	0.13 *	0.16 **	0.17 **	0.03	−0.27 **	−0.22 **	
Job burnout	2.55	0.57	−0.07	−0.07	−0.06	−0.11	0.25 **	0.24 **	0.17 **

Note: *N* = 323, * *p* < 0.05, ** *p* < 0.01.

**Table 3:** Regression results for direct effect model and mediation model.

Variables	Model 1 X→Y	Model 2 X→M	Model 3 M→Y	Model 4 X→M→Y	Model 5 MW→Y
**Control variable**					
Gender	−0.09	−0.07	−0.07	−0.09	−0.10
Age	−0.06	0.03	−0.07	−0.07	−0.08
Teaching age	0.02	−0.06	0.03	0.03	0.02
School type	−0.14 *	0.00	−0.14 *	−0.14 *	−0.15 *
**Independent variable**					
Workplace telepressure	0.19 ***	0.31 ***		0.15 ***	
**Mediator**					
Work-related rumination			0.19 ***	0.14 **	0.22 ***
**Moderator**					
Perceived organizational support					0.18 ***
Work-related rumination ∗ Perceived organizational support					−0.12 **
R^2^	0.09	0.11	0.08	0.12	0.17
F	6.39 ***	8.08 ***	5.53 ***	7.02 ***	9.09 ***

Note: *N* = 323, * *p* < 0.05, ** *p* < 0.01, *** *p* < 0.001.

**Table 4:** Bootstrap test for the mediating effect.

	Effect	BootSE	BootLLCI	BootULCI
Direct effect	0.15	0.04	0.07	0.23
Indirect effect	0.04	0.02	0.01	0.08

**Table 5:** Bootstrap test for the moderation effect.

	Effect	BootSE	BootLLCI	BootULCI
Low POS (mean − 1 SD)	0.32	0.05	0.21	0.42
POS (mean)	0.22	0.04	0.14	0.30
High POS (mean + 1 SD)	0.11	0.06	0.00	0.23

Note: *N* = 323; POS = perceived organizational support.

**Table 6:** Bootstrap test for moderated mediation model.

	Effect	BootSE	BootLLCI	BootULCI
Low POS (mean − 1 SD)	0.08	0.03	0.03	0.14
POS (mean)	0.05	0.02	0.02	0.09
High POS (mean + 1 SD)	0.02	0.02	−0.01	0.06

Note: *N* = 323; POS = perceived organizational support.

## Data Availability

The data shown in this research are available on request from the corresponding author.
